# *Escherichia coli* Phylogenetic and Antimicrobial Pattern as an Indicator of Anthropogenic Impact on Threatened Freshwater Mussels

**DOI:** 10.3390/antibiotics12091401

**Published:** 2023-09-03

**Authors:** Simone Varandas, Conceição Fernandes, Edna Cabecinha, Sónia Gomes, Gabriela Jorge da Silva, Maria José Saavedra

**Affiliations:** 1 CITAB-Inov4Agro—Centre for the Research and Technology of Agro-Environmental and Biological Sciences—Institute for Innovation, Capacity Building and Sustainability of Agri-Food Production, University of Trás-os-Montes and Alto Douro (UTAD), 5000-801 Vila Real, Portugal; simonev@utad.pt (S.V.); edna@utad.pt (E.C.); 2 CIBIO/InBIO/BIOPOLIS—Research Center in Biodiversity and Genetic Resources, University of Porto, Campus Agrário de Vairão, 4485-661 Vairão, Portugal; 3 CIMO—Centro de Investigação de Montanha/SusTEC—Laboratório Associado para a Sustentabilidade e Tecnologia em Regiões de Montanha, IPB-Institute Polytechnique of Bragança, Campus de Santa Apolónia, 5300-253 Bragança, Portugal; conceicao.fernandes@ipb.pt; 4 A2BUnit—Antimicrobials, Biocides & Biofilms Unit, Department of Veterinary Sciences, University of Trás-os-Montes and Alto Douro (UTAD), 5000-801 Vila Real, Portugal; sagomes@icbas.up.pt; 5 CIIMAR/CIIMAR-LA—Interdisciplinary Centre of Marine and Environmental Research, Novo Edifício do Terminal de Cruzeiros do Porto de Leixões, 4450-208 Matosinhos, Portugal; 6 Center for Neuroscience and Cell Biology (CNC), Faculty of Pharmacy, University of Coimbra, 3000-548 Coimbra, Portugal; gjsilva@ci.uc.pt; 7 CECAV—Animal and Veterinary Research Center and AL4AnimalS, University of Trás-os-Montes and Alto Douro (UTAD), 5000-801 Vila Real, Portugal

**Keywords:** *Escherichia coli*, One Health-EcoHealth, antimicrobial resistance, *Potomida littoralis*, *Margaritifera margaritífera*, ecological integrity

## Abstract

Freshwater bivalves are widely used as accumulation indicators and monitoring tools for assessing contaminant effects on different levels of biological integration. This pilot study aimed to explore the phylogenetic diversity of *Escherichia coli* isolated from freshwater mussels (*Margaritifera margaritifera* and *Potomida littoralis*) and characterize their phenotypes and antibiotic resistance profiles. Samples were collected in the Rabaçal and Tua Rivers, in the Douro basin, Portugal—two sites representing different levels of anthropogenic contamination. Antimicrobial susceptibility testing was performed via the disk diffusion method with 21 antibiotics. Results showed that 31% of isolates were multidrug-resistant (MDR). Thus, freshwater mussels provide an effective and time-integrated approach for identifying/quantifying fecal indicators, including MDR bacteria. PCR-based assays were designed for assessing phylogenetic *E. coli* groups. Among the *E. coli* isolates, the highest prevalence (44%) was observed in group D or E, followed by group E or Clade I (25%), group A (19%), and group B1 (13%). *E. coli* isolated from *M. margaritifera* predominantly exhibited a higher prevalence of phylogroups D or E, whereas *E. coli* from *P. littoralis* showed associations with phylogroups E or clade I, B1, A, and D or E. Our results provide new insights into the phylogenetic diversity of *E. coli* in freshwater bivalves. Additionally, the findings highlight the possible linkage of phylogroups with the host species, the geographical location in the water stream, and human activity. Using *E. coli* as a bioindicator isolated from freshwater mussels helps us grasp how human activities affect the environment. This study has important implications for those interested in safeguarding water resources, especially in tackling antibiotic resistance in aquatic ecosystems.

## 1. Introduction

The freshwater bivalve mollusks, called naiads (order Unionida), are exclusive inhabitants of rivers, streams, and lagoons. With 40% of species classified as nearly threatened, endangered, or extinct, freshwater organisms are among the most vulnerable groups globally. Among them, the order Unionida is particularly at risk, facing the highest level of endangerment [1,2]. Naiads are responsible for performing relevant ecological functions such as improving water and substrate quality and are also important ecosystem engineers that provide critical habitats for other species [3]. As these freshwater mussels are suspension feeders, they actively filter, retain, and concentrate particles from their surrounding water, including free-living or particle-bound bacteria, thus making them excellent bioindicators of water quality and contamination. In this way, mussels can reflect the load of *Escherichia coli* present in the water column at a given location. Therefore, naiads could be good candidates for studies on resistance in bacteria originating from several sources, including humans and animals, as well as providing the possibility of comparison on temporal and spatial changes [4,5]. *E. coli* has been recognized as a contributor to the dissemination of antibiotic-resistance genes in natural environments [6]. While most *E. coli* strains found in the large intestine of humans and other warm-blooded animals are considered commensal, it’s important to note that certain opportunistic and pathogenic strains have the potential to cause infections [6]. Remarkably, *E. coli* can persist in aquatic habitats due to its outstanding genetic plasticity and even multidrug resistance (MDR) [7].

The extensive use and abuse of antibiotics in anthropogenic activities, plus limited sewage treatment capacity, have resulted in increased antibiotic pollution in the environment and the emergence of antibiotic-resistant bacteria, with serious consequences for public health. Thus, in recent years, antibiotic resistance reservoirs have shifted from a narrow (clinical setting) to a more holistic approach that includes the natural environment [6,8]. The increased demand for new molecules from a clinical standpoint, both for human and animal use, coupled with the rise in antibiotic resistance, has led to increased attention to understanding the origin and tipping point of antibiotic resistance. This attention aims to curtail its dissemination and prolong the effectiveness of the remaining antibiotics [9,10]. Freshwater ecosystems are then recognized as mediators for the evolution and dissemination of antibiotic resistance and can provide a source of transferable genetic elements to human commensal bacteria and pathogens [4,11,12]. The impact of antibiotics used on humans and livestock causes constant concern and selective pressure. Although wastewater treatments have been broadly implemented, these infrastructures are not fully efficient in removing antibiotic-resistant bacteria [8,13].

The World Health Organization has identified antibiotic resistance as a paramount threat to humanity in the 21st century. Martínez [14] stated that an increase in the concentration of antibiotics in ecosystems influences antibiotic-resistant organisms and microbial population dynamics in different natural environments. The One Health concept, defined by the One Health Commission, combined with the ecosystem approach to human health, aims to consider the interactions of health, ecosystems, and society (EcoHealth). Taking this approach aims to preserve the continued efficiency of the already existing antibiotics by eliminating their inappropriate use and restoring regulation, surveillance, infection control, and sanitation [15].

The term diversity is an important and inseparable part of every healthy ecosystem, including microbial communities. Microbial diversity is the outcome of mutations, genetic recombination, and/or natural selection. Phylogenetic diversity is a measure of biodiversity based on phylogeny that provides information at the trait or species characteristics level, linking phylogeny to trait variation [16,17].

To our knowledge, this is the first study using the Clermont multiplex PCR method for the categorization of *E. coli* phylogroups isolated from threatened freshwater mussels in the Douro basin. To achieve this aim, detailed knowledge of the antibiotic resistance pattern of the *E. coli* isolated from the two mussel species and the phylogenetic diversity of *E. coli* isolates was exploited. This pilot study aimed to investigate the potential use of freshwater mussels in the Douro basin as a surveillance method for antimicrobial-resistant (AMR) organisms.

## 2. Results

### 2.1. Bacterial Isolates

**Figure 1:**
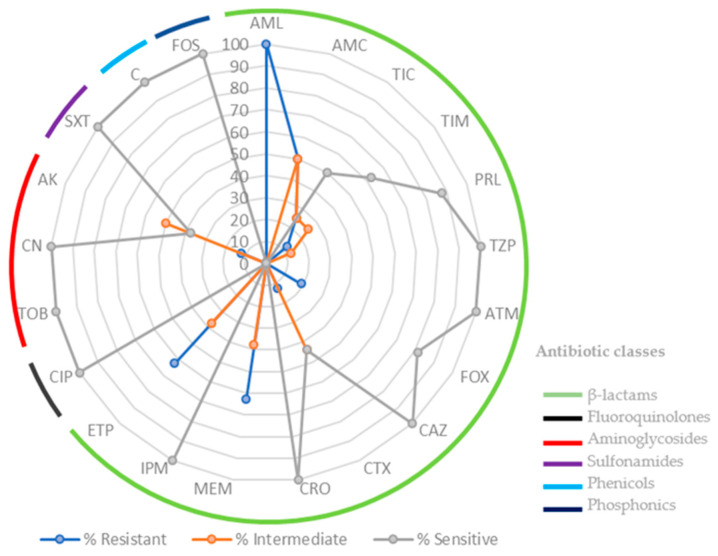
Susceptibility and resistance profiles (%) of *E. coli* (n = 16) isolates to 21 antibiotics: 14 β- lactams (AML—amoxicillin; AMC—amoxicillin/clavulanic acid; TIC—ticarcillin; TIM—ticarcillin/clavulanic acid; PRL—piperacillin; TZP—piperacillin/tazobactam; FOX—cefoxitin; CAZ—ceftazidime; CTX—cefotaxime; CRO—ceftriaxone; ATM—aztreonam; MEM—meropenem; IPM—imipenem; ETP—ertapenem); fluoroquinolones (CIP—ciprofloxacin); aminoglycosides (TOB—tobramycin; CN—gentamicin; AK—amikacin); sulfonamides (SXT—trimethoprim-sulfamethoxazole); phenicols (C—chloramphenicol); and phosphonics (FOS—fosfomycin).

In this study, 16 isolates were obtained from freshwater mussels collected from the Douro River basin, specifically 10 isolates from the 4 mussels *P. littoralis* (Tua River) and 6 isolates from the 3 mussels *M. margaritifera* (Rabaçal River). The resistance profile of each isolate was analyzed against 21 antibiotics. Multidrug resistance (MDR) is defined as acquired non-susceptibility to at least one agent in three or more antimicrobial categories [7]. The percentage of antimicrobial resistance of 16 E. coli isolates is detailed in Figure 1.

Remarkably, all the isolates showed resistance to β-lactam, with 100% of isolates demonstrating resistance against amoxicillin (AML). Isolates obtained from *P. littoralis* exhibited higher levels of antimicrobial resistance compared to those from *M. margaritifera*. Consistently, the highest incidence of resistance was to β-lactam antibiotics, namely penicillins and carbapenems (meropenem and ertapenem). *E. coli* isolates from *P. littoralis* stand out for amoxicillin/clavulanic acid (AMC—50%; 5/10), ticarcillin (TIC—40%; 4/10), meropenem (MEM—70%; 7/10), ertapenem (ETP—60%; 6/10) and aminoglycoside (amikacin AK—20%; 2/10). However, in relation to *E. coli* isolates from *M. margaritifera*, the following was observed: amoxicillin/clavulanic acid (AMC—50%; 3/6), cefoxitin (FOX—50%; 3/6), meropenem (MEM—50%; 3/6), and ertapenem (ETP—67%; 4/6). Generally, the susceptibility test revealed that 31% of the *E. coli* isolates demonstrated multidrug resistance (MDR).

### 2.2. Molecular Characterization of E. coli Isolates

Sixteen *E. coli* isolates were categorized into phylogroups according to the Clermont phylotyping method [17]. According to the classification method, the order of the phylogroups’ prevalence was as follows (Table 1): D or E, 44% (7/16); A, 19% (3/16); E or clade I, 25% (4/16); and B1, 13% (2/16).

In terms of spatial patterns, at the upstream T1 site, which experiences lower anthropogenic disturbance, *E. coli* phylogenetic analysis of *M. margaritifera* indicated the prevalence of phylogroup D or E. Conversely, downstream, where pollution levels rise, mussels (*P. littoralis*) exhibited greater diversity in *E. coli* phylogenetics, including phylogroups A, B1, D, E, and Clade I.

## 3. Materials and Methods

### 3.1. Study Area and Freshwater Mussel Sampling

**Figure 2:**
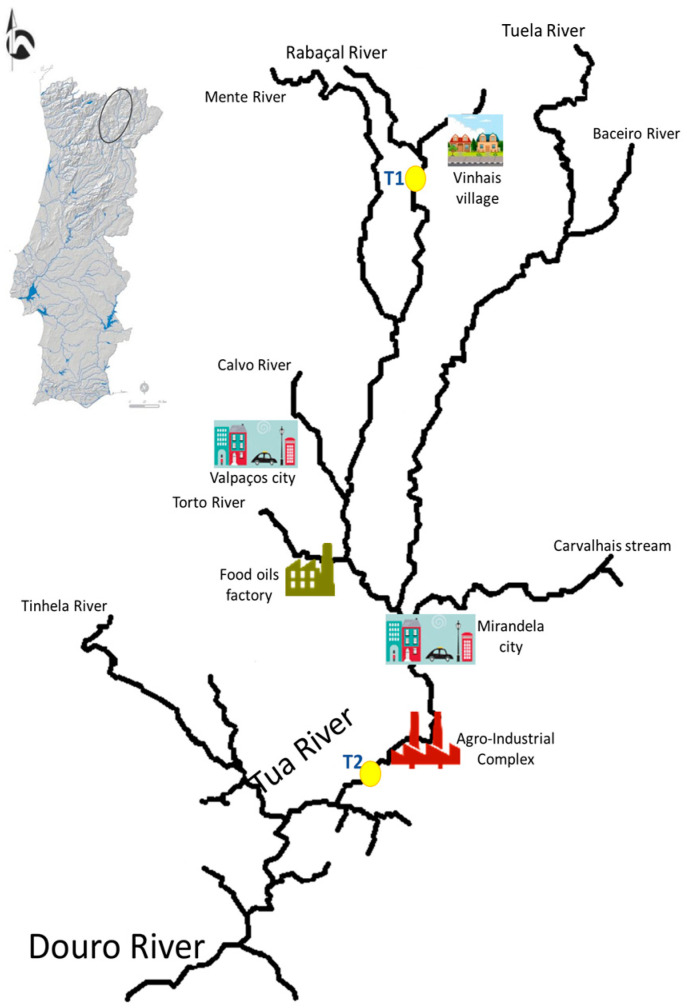
Geographical location of the study area and location of the two sampling sites (T1 and T2) [18].

The freshwater mussels were collected in Rabaçal River—T1 (3 for *M. margaritifera*, 9.93 ± 0.40 cm) and Tua River—T2 (4 for *P. littoralis*, 9.80 ± 0.29 cm), Portugal. These two sites represent a gradient of anthropogenic contamination evidenced in Figure 2; namely, the T2 site is subject to relatively high organic loads coming from agriculture, urban agglomerations (T2 site is influenced by the cities of Valpaços and Mirandela, and Vinhais village), and industrial activities (factories producing and processing food oils and others set up at an agro-industrial complex—wool, nuts, and a regional slaughterhouse), compared to the T1 site, where this is the only small village (Vinhais) upstream. Freshwater mussels were collected under the authority of a permit issued by the competent authority (Institute for the Conservation of Nature and Forest (ICNF)) and transported to the Antimicrobials, Biocides & Biofilms Unit, University of Trás-os-Montes and Alto Douro, Portugal. No ethics committee approval was needed, as we took into account the European and national legislation that refers to the creation, supply, and use of animals for scientific purposes (Directive No. 2010/63/EU, of the European Parliament and the Council, of 22 September 2010, transposed into national law through Decree-Law No. 113/7 August 2013, with the changes introduced by Decree-Law No. 1/10 January 2019). It should be highlighted that the number of organisms captured for the study was restricted to the smallest possible number because these are considered endangered species according to the IUCN Red List.

### 3.2. Bacterial Isolates

Each mussel, after being anesthetized, was aseptically opened using sterile knives, and soft tissues were collected, weighed, and diluted in buffered peptone water (1 g/9 mL) in sterile stomacher bags and homogenized for 1 min. Ten-fold serial dilutions were made up to 10^−3^ dilutions in the same diluent/saline solution, and 0.5 mL inoculum from the 10^−1^ and 10^−2^ dilutions were streaked onto the same selective and chromogenic media. In this study, Chromocult^®^ Coliform Agar (CCA) (Merck, Germany) was used to obtain *E. coli* isolates. The bacterial isolates were identified as *E. coli* by a standard microbiological detection system (VITEK^®^ 2 Compact, BioMérieux, Porto, Portugal). From preliminary isolation in CCA, pure cultures were obtained and stored at −80 °C.

### 3.3. Kirby-Bauer Disk Diffusion Susceptibility Test

The antimicrobial susceptibility testing (AST) was performed using the disk diffusion method (Kirby–Bauer) on Muller–Hinton agar (Merck, Germany) based on the Clinical and Laboratory Standards Institute (CLSI, 2022). Medium Muller–Hinton agar (MH) (Merck, Germany) was prepared according to the manufacturer’s instructions. From the pure cultures previously obtained, three to four colonies were retrieved and suspended in saline solution, with turbidity adjusted to the 0.5 McFarland standard (approx. cell density 1.5 × 10^8^ CFU/mL), according to the Performance Standards for Antimicrobial Susceptibility Testing. The inoculum was then evenly spread across the whole medium surface. Isolates were tested against 21 antibiotic disks. The β-lactam antibiotics tested included penicillin (aminopenicillins, carboxypenicillins, and ureidopenicillins), cephalosporins (2nd and 3rd generations), and monobactams and carbapenems, namely amoxicillin (AML), amoxicillin/clavulanic acid (AMC), ticarcillin (TIC), ticarcillin/clavulanic acid (TIM), piperacillin (PRL), piperacillin/tazobactam (TZP), cefoxitin (FOX), ceftazidime (CAZ), cefotaxime (CTX), ceftriaxone (CRO), aztreonam (ATM), meropenem (MEM), imipenem (IPM), and ertapenem (ETP). Additionally, another 6 classes of antibiotics were tested: fluoroquinolones (ciprofloxacin—CIP), aminoglycosides (tobramycin—TOB, gentamicin—CN, amikacin—AK), fosfomycin (FOS), chloramphenicol (C), and the combination trimethoprim-sulfamethoxazole (SXT). All the antibiotics were conserved at 4 °C at predefined concentrations. *E. coli* ATCC 25922 was used as the control strain. The disks were applied to the culture medium surface with a dispenser. Plates were incubated at 35 ± 1 °C for approximately 24 h. Afterwards, isolates were classified as sensitive (S), intermediate (I), or resistant (R) based on the size of the growth inhibition zone of the bacteria, according to CLSI guidelines.

### 3.4. Molecular Characterization of E. coli Isolates

Sixteen *E. coli* isolates were obtained from the original collection. Total DNA was extracted from one single colony by using the GF-1 Bacterial DNA Extraction Kit (Vivantis, Malasia), according to the manufacturer’s instructions. The quality and quantity of the extracted DNA were assessed using measurements (A260 nm/A280 nm and A260 nm/A230 nm) on a NanodropTM 1000 Spectrophotometer (Nanodrop Technologies, Wilmington, DE, USA) and electrophoresis on 0.8% agarose in 1× TAE buffer (Tris-acetate EDTA). The extracted DNA was utilized as a template for identifying phylogroups of *E. coli* isolates. The phylogroups were determined using the quadruplex PCR assay [17].

A set of specific primers were synthesized by STAB Vida (Portugal) according to primer sequences described in the extended quadruplex method for genotyping *E. coli* isolates, following the approach outlined by Clermont et al. [17]. The phylogenetic groups were differentiated by the presence or absence of the *chuA*, *yjaA*, *TspE4*.*C2*, and *arpA* genes (Table 2). In cases of uncertainty between phylogroups A or C, screening was performed using specific primers; similarly, specific primers were used for discriminating between phylogroups D or E. Each PCR reaction was carried out in a total volume of 20 μL, containing 2 μL of genomic DNA (at approximately 100 ng/μL), 10× PCR buffer with (NH_4_)_2_SO_4_, 2 μM each dNTP, 2 U of Taq DNA polymerase (Thermo Fisher Scientific, Waltham, MA, USA), and 10 μM of each forward and reverse primer. Amplifications were conducted in a 96-well plate thermocycler (Applied Biosystems, Foster City, CA, USA), with the following PCR cycle: 95 °C for 4 min, followed by 30 cycles of 94 °C/5 s, 57 °C/1 min (group E) or 59 °C/1 min (quadruplex and group C), 72 °C/1 min, and a final step of 72 °C for 5 min. The PCR products were separated using electrophoresis on 1.5% agarose gels in 1× TAE buffer (Tris-acetate EDTA) with SYBR^®^Safe DNA Gel Stain (Thermo Fisher Scientific).

## 4. Discussion

Bivalves are excellent filter feeders and have a high filtration capacity, making them excellent bioindicators of water quality [1,18,19]. It has been shown that they can accumulate heavy metals [20] as well as bacteria [18,21], providing information on the environmental impact of the ecosystem. Our results showed that *E. coli* isolates from freshwater mussels were resistant to the last available resource antibiotics (carbapenems: meropenem—MEM and ertapenem—ETP, used exclusively in hospitals), which precedes a “One Health” problem. Among β-lactams, carbapenems are considered the most effective antibiotics against both gram-positive and gram-negative bacteria. As carbapenems are highly effective and less vulnerable to β-lactamases, they are the most trustworthy last-resort treatment for bacterial infections. For these reasons, the spread of carbapenem resistance constitutes a global public health problem of extreme importance [22].

The isolates from *P. littoralis* species (T2), which were collected downstream from multiple sources of contamination input (Figure 2), showed resistance to ticarcillin, ticarcillin/clavulanic acid, meropenem, ertapenem, and amikacin, while the isolates from the upstream site, which included *M. margaritifera* species (T1), showed resistance to cefoxitin, meropenem, and ertapenem (Figure 1). It can therefore be deduced that, at T2, the samples have a higher percentage of antimicrobial resistance due to the greater influence of anthropogenic impact in the area, such as the presence of more industry, a greater population, and higher pressures related to livestock farms. In addition, T2 is downstream from wastewater treatment plant facilities, which have been recognized as a source of antimicrobial-resistant bacteria [23]. Antibiotics and their metabolites reach wastewater treatment plants through human and/or animal excretion. These compounds and bacteria are not removed during the wastewater treatment process, and, as a result, they are eventually released into aquatic streams [24,25].

One of the main risks for public health is contaminated water, which can act as a reservoir for resistance genes that can be transferred from environmental bacteria to human pathogens and commensals [26]. Our results showed that 31% of the *E. coli* strains isolated from the two freshwater mussel species were resistant to three antibiotic classes. Wambugu et al. [23] found, in *E. coli* isolates from river water, 65% showed resistance to three or more classes of antimicrobials, while 55% showed resistance to four or five antimicrobials, which is above the results reported here. According to Anjum et al. [27], antimicrobial-resistant *E. coli* is an indicator of the levels and anthropogenic influences of MDR in the environment. The presence of high MDR strains has been associated with industrial effluents as well as lands with livestock pressure or high anthropogenic pressure [28]. The findings obtained from our study should be seen as a warning of a potential risk to the population in the vicinity of the study area, which still uses fish and river bivalves in their daily diet. The development and spread of antibiotic resistance among bacteria affecting human health, such as *E. coli*, is one of the main problems worldwide [29]. The genetic determinants accountable for conferring resistance are often carried by transmissible mobile elements (conjugative plasmids, gene cassettes in integrons, and transposons) that are able to be transferred between bacterial cells, resulting in the transmission of resistance to other strains and species [30].

Understanding the phylogenetic diversity of *E. coli* isolates obtained from freshwater mussels is a fundamental objective to determine the type of impact on the ecological integrity of the water systems. Here, the study area comprises the Rabaçal and Tua Rivers, tributaries of the River Douro basin, Portugal. Although this study is the first to approach the genetic diversity of *E. coli* strains isolated from two freshwater bivalves and their antibiotic resistance profile, it is paramount to determine the impacts on freshwater mussels’ ecological integrity to predict and prevent the spread of *E. coli* strains. Therefore, bacterial indicators assume special importance in surveillance schemes that identify emerging risks as a priority for public health, considering the EcoHealth concept. This work provides an overview of anthropogenic impacts on the studied rivers (ecological niches: water and bivalves) as reservoirs of multi-resistant bacteria, which can be applied to other rivers. The use of two freshwater mussel species listed as threatened in the IUCN Red List (*Margaritifera margaritifera* and *Potomida littoralis*) as bioindicators will allow a better understanding of the effects of anthropogenic stressors on the study area.

The results are consistent with the phylogroups obtained since *arpA* yield is expected in all *E. coli* and clade I strains, except for strains belonging to phylogroups B2 and F [17]. Group D or E were the most highly prevalent isolates (44%) (7/16), followed by group E or Clade I, with 25% (4/16). Group E or Clade I were associated with the greatest number of MDR isolates. Clermont et al. [17] showed that strains responsible for extra-intestinal infections were much more likely to belong to phylogroups C, B2, or D rather than A or B1, which generally lack a distinct virulence profile and are classified as commensal strains. Several studies report a higher prevalence of *E. coli* phylogroups A and B1 in mollusks [30,31,32], with A being dominant, and the results achieved in this study indicate that 19% of the samples belong to phylogenetic group A, while 13% belong to B1.

Despite the limited number of isolates used in this study due to official restrictions on collecting freshwater mussels, results show high diversity in phylogenetic distribution, both between the two sampling sites and in resistance to the antimicrobials tested. Regarding spatial trends, at the upstream T1 site with less anthropogenic disturbance, the *E. coli* from *M. margaritifera* was characterized as phylogroup D or E. Downstream, the pollution gradient increased, and *P. littoralis* presented a higher *E. coli* phylogenetic diversity (A, B1, D or E, or Clade I). It is unclear whether these differences in phylogroup distribution can be attributed to geographical factors, i.e., anthropogenic disturbance, or host factors, i.e., different species, or a combination of both. Further studies should be done to understand the ecology of bacteria in aquatic environments.

According to the World Health Organization, tackling antibiotic resistance should be a priority. Furthermore, this analysis requires a holistic One Health approach involving humans, animals, and the environment. Antibiotic-resistant bacteria, once disseminated in the environment, survive and proliferate by reaching new habitats and, consequently, the food chain. Bacterial resistance to antimicrobials occurring in food-aquatic animals can spread to humans via food-borne routes. This has been observed for the zoonotic bacteria *E. coli*, both through routes such as water or other environmental contamination and through direct contact with animals [33]. Therefore, acting on the environment is key to mitigating the impact and risk that this problem causes in society. Knowledge of the presence of pollutants in water and how they affect the ecosystem and, consequently, human health is a challenge due to the limited and difficult monitoring of these compounds. However, the use of bioindicators such as freshwater mussels is widespread and has produced excellent results [34,35,36].

The applicability of freshwater mussels as indicators for antimicrobial resistance (AMR) is a relatively emerging research area, and further investigations are necessary to validate their efficacy comprehensively. Additionally, it is crucial to account for variables such as variations in mussel feeding behavior. In this study, we encountered some limitations, including the challenges of collecting a limited number of mussels due to restrictions. The use of bivalves may be limited due to stress factors that induce species decline. This may be important for endangered or protected populations such as *M. margaritifera*. This species has several life history traits (e.g., long life span, delayed reproduction, and low juvenile survival) that make them highly susceptible to impacts generated by anthropogenic activities [37]. This high susceptibility can explain why we only found it at the upstream point, the site with the lowest anthropogenic pressure.

The impacts on water ecological integrity must be assessed from a multidisciplinary perspective, integrating multiple competencies and perspectives [38,39]. In addition, these results highlight the need to integrate microbiological analyses into aquatic ecosystem monitoring, considering interactions between geographical and ecological elements, human activities, and agriculture components within the “EcoHealth” approach. Finally, it should be noted that, according to the Interagency Coordination Group on Antimicrobial Resistance, antibiotic resistance represents a global public health challenge and contributes to impeding the achievement of the Sustainable Development Goals (SDGs) of the 2030 Agenda related to health, food security, clean water, and sanitation.

## 5. Conclusions

Although a specific ecosystem, the resistance patterns of *E. coli* to antibiotics observed in the present study indicate that the freshwater mussels analyzed can be an effective bioindicator organism for monitoring antimicrobial resistance in rivers. There is a need for more studies regarding the phylogenetic analysis of *E. coli* strains from freshwater mussels to establish a more efficient connection between multi-resistance, phylogroups, and the source of contamination. Our findings can have management implications, leading to surveillance measurements that can be oriented towards communities, bringing a holistic perspective to the ecological integrity of aquatic ecosystems.

## Figures and Tables

**Table 1:** Distribution of different phylogroups of *E. coli* isolates form freshwater mussels with specific genes by using the quadruplex method. Mg—*M. margaritifera*; Pt—*P. littoralis*.

Isolates	*arpA* 400 bp	*chuA* 288 bp	*yjaA* 211 bp	*TspE4.C2* 152 bp	Phylogroup (%)
Pt1; Pt3; Pt4; Pt7	+	+	+	-	E or Clade I (40%)
Pt8; Pt9	+	-	-	+	B1 (20%)
Pt2; Pt5; Pt6	+	-	-	-	A (30%)
Pt10	+	+	-	+	D or E (10%)
Mg1; Mg2; Mg3; Mg4; Mg5; Mg6	+	+	-	-	D or E (100%)

**Table 2:** Phylogenetic ^1^ genes with the primer sequences and expected amplicon size.

Primer Name	Primer Sequence (5′-3′)	Amplicon Size (bp)
*chuA*	F: 5-ATGGTACCGGACGAACCAAC-3 R: 5-TGCCGCCAGTACCAAAGACA-3	288
*yjaA*	F: 5-CAAACGTGAAGTGTCAGGAG-3 R: 5-AATGCGTTCCTCAACCTGTG-3	211
*TspE4C2*	F: 5-CACTATTCGTAAGGTCATCC-3 R: 5-AGTTTATCGCTGCGGGTCGC-3	152
*arpA*	F: 5-AACGCTATTCGCCAGCTTGC-3 R: 5-TCTCCCCATACCGTACGCTA-3	400
*arpA* (group E)	F: 5-GATTCCATCTTGTCAAAATATGCC-3 R: 5GAAAAGAAAAAGAATTCCCAAGAG	301
*pA* (group C)	F: 5-AGTTTTATGCCCAGTGCGAG-3 R: 5-TCTGCGCCGGTCACGCCC-3	219

^1^ Primer pairs; ArpAgpE.f, ArpAgpE.r and trpAgpC.1, trpAgpC.2 were used for the determination of groups E and C, respectively. Internal control was used in group E and C determination, as reported by Clermont et al. [17].

## Data Availability

All data related to this research are contained within this paper.
